# Cold snare and ablation technique for endoscopic mucosal resection of incompletely resected large laterally spreading tumors

**DOI:** 10.1055/a-2106-2061

**Published:** 2023-07-11

**Authors:** Roupen Djinbachian, Mahsa Taghiakbari, Mohammed El Mehdi El Yamani, Daniel von Renteln

**Affiliations:** 1 Division of Gastroenterology, Montreal University Hospital Center (CHUM), Montreal, Canada; 2 Montreal University Hospital Research Center (CRCHUM), Montreal, Canada; 3 Division of Internal Medicine, Montreal University Hospital Center (CHUM), Montreal, Canada

Previously attempted and partially resected laterally spreading tumors (PA-LSTs) present a particular challenge for subsequent endoscopic mucosal resection (EMR) owing to the presence of submucosal fibrosis. Techniques such as hot avulsion, or cold avulsion with adjuvant snare tip soft coagulation have been described previously, with local recurrence rates of 15 % 1 2 . Hot EMR is challenging for PA-LSTs given that lesions are often nonlifting, which, combined with the nonrigid soft snare, makes capturing the mucosa difficult, hampering resection of these lesions 3 . The combination of cold EMR with ablation might prove to be a good combination. The rigidity of the cold snare offers the capacity to push firmly against mucosa, allowing for improved engagement of the snare in the submucosal plane and this can be combined with enhanced submucosal injection and ablation using hybrid argon plasma coagulation (hAPC) in the COld Snare with Ablation (COSA) technique 4 .

We present a case of COSA for a 25-mm nonlifting PA-LST ( Video 1 ). Initial submucosal lifting was performed using needle injection and dynamic lifting, which achieved only partial lift. The lesion was subsequently resected piecemeal using a cold snare. The cold snare was pushed firmly against the mucosa to allow dissection of the submucosal plane. Additional submucosal injection was performed using the hybrid component of the hAPC probe (ERBE Elektromedizin Gmbh, Tübingen, Germany) to expand the submucosal space of the defect and allow for a safety cushion, preventing damage to the muscular layer prior to thermal margin and base ablation ( Fig. 1 ). Additional prophylactic ablation of the EMR base was performed to prevent recurrence originating from the base ( Fig. 2 ). Particular attention was paid to ablating the central nonlifting portion of the lesion using hAPC. The margins of the defect were then ablated using the same hAPC probe to create a ring of ablated mucosa surrounding the resection site ( Fig. 2 ).

**Video 1** Cold snare resection of a 25-mm colorectal tubular adenoma with prophylactic thermal ablation of the resection margin and base.


**Fig. 1:**
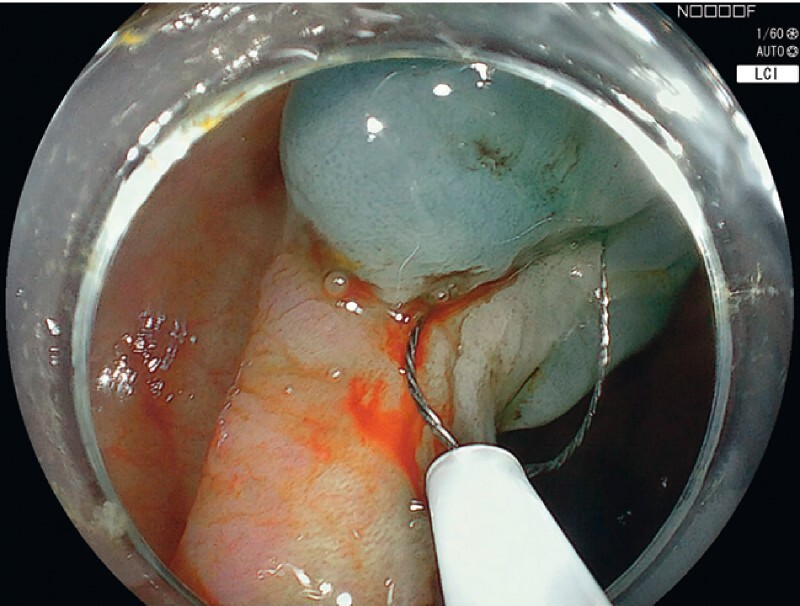
Cold snare resection of the previously attempted lesion.

**Fig. 2:**
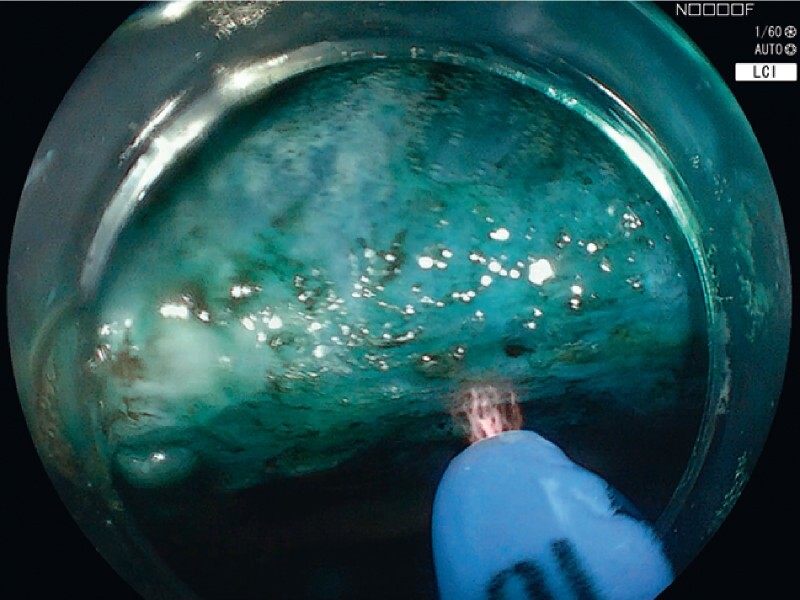
Hybrid argon plasma coagulation-assisted thermal ablation of the resection base.

Endoscopy_UCTN_Code_TTT_1AQ_2AD

## Notes

Endoscopy E-Videos

https://eref.thieme.de/e-videos
*E-Videos* is an open access online section of the journal *Endoscopy* , reporting on interesting cases and new techniques in gastroenterological endoscopy. All papers include a high-quality video and are published with a Creative Commons CC-BY license. Endoscopy E-Videos qualify for HINARI discounts and waivers and eligibility is automatically checked during the submission process. We grant 100% waivers to articles whose corresponding authors are based in Group A countries and 50% waivers to those who are based in Group B countries as classified by Research4Life (see: https://www.research4life.org/access/eligibility/ ). This section has its own submission website at https://mc.manuscriptcentral.com/e-videos
